# Thermopriming Induces Time-Limited Tolerance to Salt Stress

**DOI:** 10.3390/ijms25147698

**Published:** 2024-07-13

**Authors:** Tobias Körner, Jana Zinkernagel, Simone Röhlen-Schmittgen

**Affiliations:** Department of Vegetable Crops, Hochschule Geisenheim University, 65366 Geisenheim, Germany; tobias.koerner@hs-gm.de (T.K.);

**Keywords:** abiotic stress, *Solanum lycopersicum*, plant growth, plant development, fruit yield, climate change, food security, sustainable plant protection, benzothiadiazole

## Abstract

Implementing sustainable crop protection practices is crucial to protect global harvests and ensure high-quality food supplies. While priming is an established method in seed production for the fortification of plants against various stresses, it is not yet a standard practice in transplant cultivation. Thus, we evaluated the long-term effects of thermopriming—a heat-based priming technique—on the growth, development, and fruit yield of tomato plants. Following a recovery period of about six weeks for thermoprimed plants without stress inducers, we subjected them to subsequent salt stress to ascertain the persistence of the priming effects. Additionally, we compared the efficacy of thermopriming with benzothiadiazole (BTH), a chemical elicitor, in enhancing plant resilience to abiotic stress. While BTH application negatively impacted both plant growth and fruit health, thermopriming showed no such adverse effects on these parameters. Instead, thermopriming initially enhanced the plant defense mechanisms by increasing the accumulation of protective phenols and flavonoids in the leaves. Interestingly, while thermopriming did not alter the response to salt stress, it notably strengthened the overall resilience of the plants. Our findings underscore both the potential and temporal constraints of thermopriming memory. Nonetheless, primed plants exhibited temporarily increased stress tolerance, offering a means to safeguard the offspring.

## 1. Introduction

The global population continues to increase, according to projections from the United Nations, albeit at a seemingly slower pace [1]. It has been suggested that by attaining the Sustainable Development Goals (SDGs), the population growth rates could be moderated, offering greater control over limited resources and mitigating the vulnerability to global challenges like food insecurity [1,2,3]. Sustainable agricultural practices can contribute to enhancing global nutrition and food security by minimizing the risks of yield losses (SDG 2) [4]. Nevertheless, enhancing agricultural and horticultural food production poses significant challenges, especially on a global scale, amidst risks associated with unpredictable climate change [5]. The Intergovernmental Panel on Climate Change (IPCC) [5] has projected an increase in the frequency, intensity, and duration of heatwaves, among numerous other global challenges.

Heat considerably impacts vegetable production and product quality [6]. Additionally, salinity poses a threat to valuable agricultural land—just two among the many (a)biotic stressors that challenge plant production globally [7,8]. Hence, a sustainable approach in plant production becomes crucial to secure crop yields, including those of tomatoes. In this context, priming shows potential in enhancing plant protection by inducing (cross-)tolerance to various stresses [9]. Priming can be implemented at different developmental stages (e.g., seeds, juvenile, and mature plants) and in response to various stresses, such as heat through ‘thermopriming’ [10,11]. However, this natural adaptation imposes physiological costs for both vegetative and generative plant growth, as reported in previous studies [12,13]. Nonetheless, (thermo-)priming enhances plants’ ability to better cope with subsequent and recurring stressors [14].

In this context, several studies have demonstrated the beneficial effects of structural analogues to salicylic acid (SA), such as benzothiadiazole (BTH; also known as acibenzolar-S-methyl, ASM), in inducing systemic acquired plant resistance (SAR) against biotic stress [15,16,17,18]. SA plays a crucial role as a signaling molecule in activating plant defense responses [19,20]. While Yasuda et al. [21] reported an antagonistic interaction between SAR and the abscisic-acid-mediated abiotic stress response, Zandalinas et al. [22] demonstrated the upregulation of SA following heat stress. Hence, we incorporated the BTH treatment into our experiment as an inducer of SAR, alongside thermopriming preconditioning, to explore their interaction with subsequent salt stress and their impact on (i) vegetative and generative plant growth and development and (ii) the accumulation of primary and secondary metabolites in young and mature tomato leaves and fruits. However, the primary focus of this study centered on the effects of thermopriming as a preconditioning measure for transplants to enhance their stress tolerance during cultivation. In synergy with the research questions addressed in another study [23], we sought to validate whether thermopriming still confers cross-tolerance to salinity after an extended recovery period between treatments. These insights can be used for the evaluation of thermopriming as a strategy to cultivate plants with natural stress adaptation, in contrast to the use of chemical elicitors like BTH, which may also trigger systemic resistance. Ultimately, securing plant yields and the food supply is essential for the global implementation of the Sustainable Development Goals (SDGs). Thus, we present the thermopriming of transplants as a potential practice to safeguard plant production.

## 2. Results

### 2.1. Vegetative Plant Growth and Development

**Figure 1:**
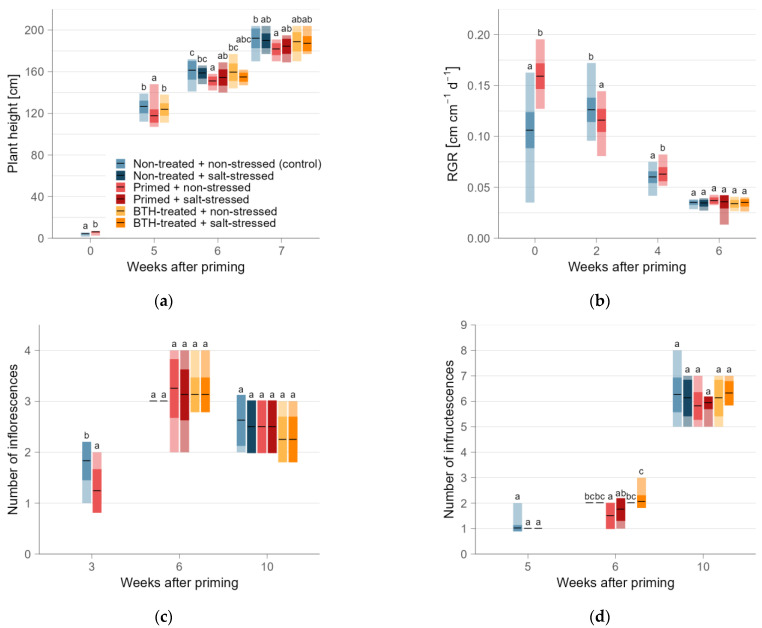
Growth parameters of tomato plants, displayed by mean (black horizonal line), standard deviation (intensely colored inner box), and maximum and minimum (lightly colored outer box), summarized for weeks after priming, differentiated for the six treatments: non-treated and non-stressed (control; light blue), non-treated and salt-stressed (dark blue), primed and non-stressed (light red), primed and salt-stressed (dark red), BTH-treated and non-stressed (yellow), and BTH-treated and salt-stressed (orange). (**a**) Plant height; (**b**) RGR (relative growth rate); (**c**) number of inflorescences; (**d**) number of infructescences. The different letters indicate significant differences (ANOVA and EMMs post hoc; α = 0.05) between groups at the same week after priming. The initial sample size after priming for plant height was n = 277 (n = 273 for RGR) for non-primed (control) and n = 139 (n = 138 for RGR) for primed plants. Then, at 2 WAP, the sample size decreased to n = 224 for non-primed and n = 112 for primed plants and, at 3 and 4 WAP, to n = 192 (non-primed)/n = 96 (primed) plants per treatment. At 5 WAP, the sample size for all parameters was n = 64 plants per treatment and, after that, from 6 WAP, it was n = 16 plants per treatment.

In response to thermopriming, primed plants initially exhibited an increased plant height (+46%; Figure 1a). However, in the subsequent weeks, primed plants showed a decrease in plant height (up to −12%). Even after the application of BTH, thermoprimed plants continued to display a reduced plant height compared to BTH-treated and non-primed control plants (−7%). The subsequent salt stress at WAP 6 did not change this trend among the treatments. Both non-primed and BTH-treated plants maintained an increased height compared to primed plants. Notably, the control group, comprising non-primed and non-stressed plants, exhibited the highest increase in plant height. Although non-primed plants did not differ in height from other treatments after the subsequent stress, primed plants exhibited a decreased height compared to control plants, with the most pronounced reduction observed in the following weeks. Neither the BTH treatment nor the salt stress had a significant effect on the plant height. Throughout the experiment, thermoprimed plants consistently displayed a decreased height, except immediately after priming. Consistent with the height observations, we overall noted decreased internode lengths in primed plants, approximately 7% less than the control group.

Following priming, we observed an increase in RGR (+50%) in thermoprimed plants compared to non-primed control plants (Figure 1b). However, this effect was reversed two weeks later (−8%). Subsequently, thermoprimed plants exhibited an increased RGR, while the BTH treatment showed no effect on growth. After the salt stress at 6 WAP, the RGR did not differ between treatments.

**Figure A1:**
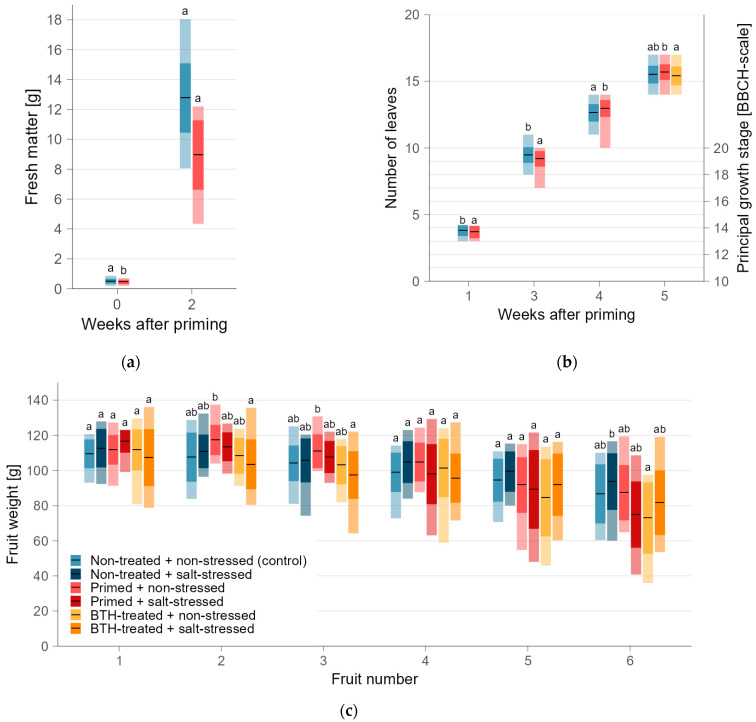
Vegetative and generative growth parameters of tomato plants, displayed by mean (black horizonal line), standard deviation (intensely colored inner box), and maximum and minimum (lightly colored outer box), summarized for weeks after priming, differentiated for the six treatments: non-treated and non-stressed (control; light blue), non-treated and salt-stressed (dark blue), primed and non-stressed (light red), primed and salt-stressed (dark red), BTH-treated and non-stressed (yellow), and BTH-treated and salt-stressed (orange). (**a**) Fresh matter; (**b**) number of leaves; (**c**) single fruit weight of early infructescences (ascending number of fruits by decreasing fruit age). The different letters indicate significant differences (ANOVA and EMMs post hoc; α = 0.05) between groups at the same week after priming and fruit number, respectively. The initial sample size after priming for fresh matter was n = 46 for non-primed (control) and n = 26 for primed plants. At 2 WAP, the sample size for fresh matter decreased to n = 32 for non-primed and n = 16 for primed plants. For the number of leaves, the sample size per treatment was as follows: n = 224 (non-primed)/n = 112 (primed) at 1 WAP, n = 192 (non-primed)/n = 96 (primed) at 3 and 4 WAP, and n = 64 at 5 WAP. For the single fruit weight, the sample size for each number of fruits differed between n = 46 and 48 per treatment.

Thermopriming initially delayed vegetative plant growth in the first three weeks, with thermoprimed plants temporarily displaying a higher number of leaves at WAP 4, indicating accelerated plant development in regard to the plant growth stage (Figure A1b). However, no differences in growth stage were observed between primed and control plants in the following weeks, whereas BTH-treated plants exhibited a significantly lower number of leaves than thermoprimed plants at 5 WAP.

Regarding generative plant development, thermopriming initially resulted in a lower number of inflorescences per plant compared to the non-primed control (−32%; Figure 1c). However, neither the BTH treatment nor the subsequent salt stress affected the inflorescences. After the salt stress application, the treatments did not differ in inflorescences. Initial differences in infructescences between treatments were only observed after BTH application (Figure 1d). Following the subsequent salt stress, thermoprimed plants displayed delayed fruit development, with 25% fewer infructescences in primed plants and 12% fewer in primed and stressed plants compared to the control. This effect persisted until 10 WAP, after which the fruit development among treatments aligned.

After thermopriming, no differences were observed between treatments in terms of the total FM, although, at 2 WAP, primed plants exhibited decreased FM compared to non-primed control plants (Figure A1a). By the end of the experiment, neither the total FM nor the FM of the stems and leaves separately differed between treatments, which was consistent with the DM findings (Table A1).

**Figure A2:**
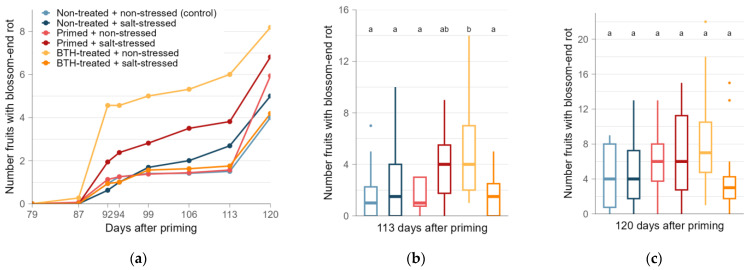
Accumulated number of non-marketable fruits of tomato plants with blossom-end rot, displayed by (**a**) trend lines representing means specific to each treatment and each harvest date in days after priming and (**b**) for 113 days after priming, as well as (**c**) for 120 days after priming as boxplots, differentiated for the six treatments (from left to right): non-treated and non-stressed (control; light blue), non-treated and salt-stressed (dark blue), primed and non-stressed (light red), primed and salt-stressed (dark red), BTH-treated and non-stressed (yellow), and BTH-treated and salt-stressed (orange). The different letters indicate significant differences (ANOVA and EMMs post hoc; α = 0.05) between groups. The sample size over the whole experimental duration of all harvested plants per date was n = 16, although, at the first two dates (79 and 87 days after priming, respectively), the overall sample size was lower due to delays in harvest between plants.

No differences were observed in the fruit yield among the treatments over the entire harvest period compared to the control (Table A2). However, due to the BTH application, we noted a decrease in the size of individual fruits, up to 4% smaller than the control (Figure A1c). In particular, the second, third, and sixth fruits were affected during the early stages of harvest, while the sizes of the first, fourth, and fifth fruits per truss did not differ among the treatments. Additionally, the fruits of BTH-treated (not stressed) plants were more susceptible to blossom-end rot, similar in trend to the thermoprimed and subsequently stressed plants (Figure A2a,b). However, by the end of our experiment, all treatments showed an equal loss of marketable fruits due to blossom-end rot (Figure A2c).

**Figure 2:**
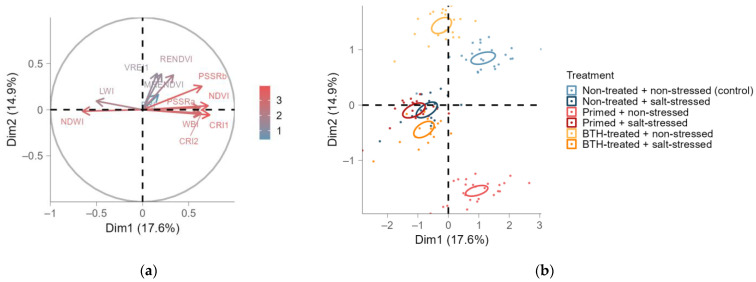
Multiple factor analysis (MFA) of standardized vegetation indices of tomato leaves for group means (specified by treatment, leaf age, and date) displayed as points for the six treatments with confidence ellipses (β = 0.95, (**b**)), i.e., non-treated and non-stressed (control; light blue), non-treated and salt-stressed (dark blue), primed and non-stressed (light red), primed and salt-stressed (dark red), BTH-treated and non-stressed (yellow), and BTH-treated and salt-stressed (orange), or displayed as arrows for input parameters (**a**). MFA was performed on the active variable treatment as well as the supplementary variables: leaf age and date. The color gradient in (**a**) indicates the contributions of the variables to the dimensions (Dim).

**Figure A3:**
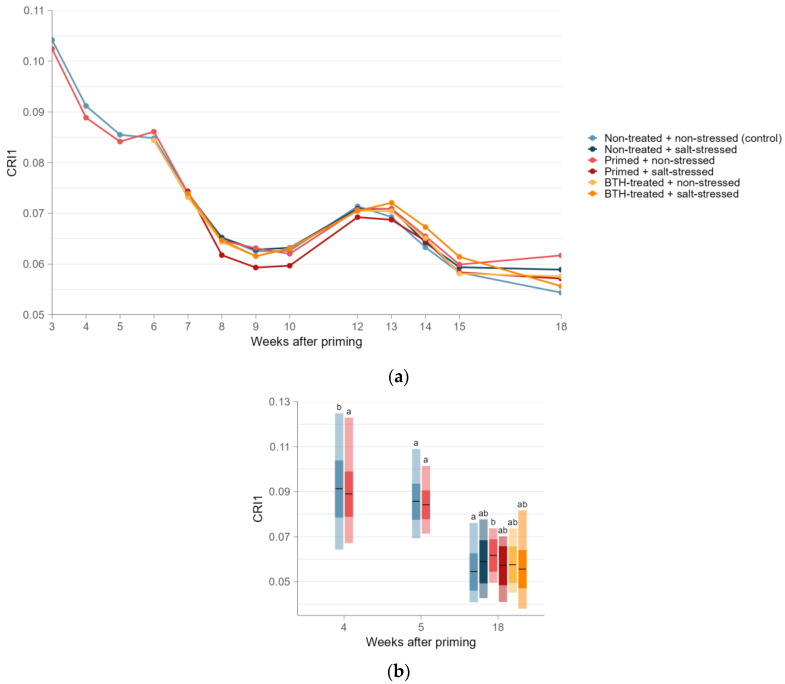
CRI1 of young and mature tomato leaves, displayed by (**a**) trend lines representing means specific to each treatment week after priming and (**b**) mean (black horizonal line), standard deviation (intensely colored inner box), and maximum and minimum (lightly colored outer box) weeks after priming, differentiated for the six treatments (from left to right): non-treated and non-stressed (control; light blue), non-treated and salt-stressed (dark blue), primed and non-stressed (light red), primed and salt-stressed (dark red), BTH-treated and non-stressed (yellow), and BTH-treated and salt-stressed (orange). The different letters indicate significant differences (ANOVA and EMMs post hoc; α = 0.05) between groups at the same week after priming. The sample size per treatment was as follows: n = 191 (non-primed)/n = 96 (primed) at 3 WAP, n = 384 (non-primed)/n = 192 (primed) at 4 WAP, n = 256 (non-primed)/n = 127 (primed) at 5 WAP, n = 64 (non-primed and BTH-treated)/n = 63 (primed) at 6 WAP, and n = 32 from 7 WAP for all treatments.

**Figure A4:**
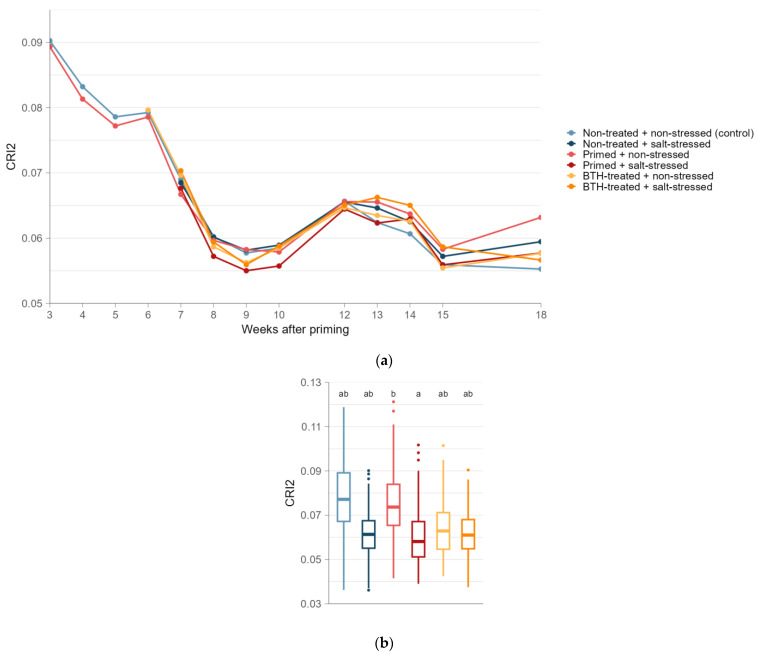
CRI2 of young and mature leaves, displayed by (**a**) trend lines representing means specific to each treatment week after priming and (**b**) boxplots over all dates, differentiated for the six treatments (from left to right): non-treated and non-stressed (control; light blue), non-treated and salt-stressed (dark blue), primed and non-stressed (light red), primed and salt-stressed (dark red), BTH-treated and non-stressed (yellow), and BTH-treated and salt-stressed (orange). The different letters indicate significant differences (ANOVA and EMMs post hoc; α = 0.05) between groups at the same week after priming. The sample size per treatment was as follows: n = 191 (non-primed)/n = 96 (primed) at 3 WAP, n = 384 (non-primed)/n = 192 (primed) at 4 WAP, n = 256 (non-primed)/n = 127 (primed) at 5 WAP, n = 64 (non-primed and BTH-treated)/n = 63 (primed) at 6 WAP, and n = 32 from 7 WAP for all treatments.

Moreover, we evaluated several vegetation indices to assess the health and stress statuses of plants post-treatment. The MFA was conducted to analyze the correlation between the VIs and their overall effects on the treatments (Figure 2b). The NDWI (positively correlated with LWI) showed a negative correlation with a group of Vis, including the NDVI, CRI1, CRI2, WBI, and PSSRa, which were positioned in the opposite quadrant and displayed a positive correlation within (Figure 2a). Apart from these groups, the PRI, RENDVI, MRENDVI, and VREI1 were clustered together and, thus, positively correlated. This group of VIs did not correlate with the first two groups and did not explain as much variability in the dimensions. Therefore, our primary focus was on the NDWI (group 1), as well as the NDVI, CRI1, and CRI2 (group 2), which explained most of the experimental variability. Regarding CRI1 (Figure A3 and Figure A4), thermopriming initially led to decreased carotenoid content but ultimately resulted in an increase compared to the control group, consistent with CRI2. However, no treatment effects were observed for both VIs after subsequent stress.

**Figure A5:**
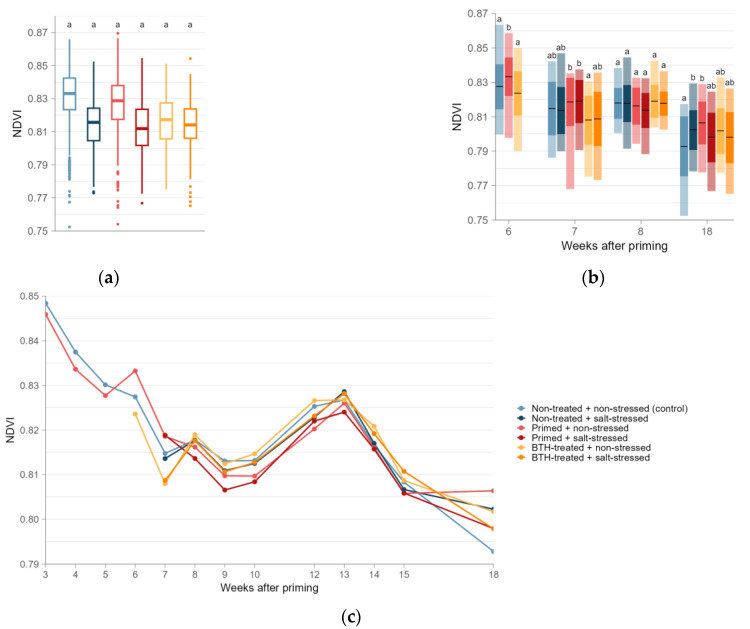
NDVI of young and mature tomato leaves, displayed by (**a**) boxplots over all dates, (**b**) mean (black horizonal line), standard deviation (intensely colored inner box), and maximum and minimum (lightly colored outer box) weeks after priming, and (**c**) trend lines representing means specific to each treatment week after priming, differentiated for the six treatments (from left to right): non-treated and non-stressed (control; light blue), non-treated and salt-stressed (dark blue), primed and non-stressed (light red), primed and salt-stressed (dark red), BTH-treated and non-stressed (yellow), and BTH-treated and salt-stressed (orange). The different letters indicate significant differences (ANOVA and EMMs post hoc; α = 0.05) between groups at the same week after priming. The sample size per treatment was as follows: n = 191 (non-primed)/n = 96 (primed) at 3 WAP, n = 384 (non-primed)/n = 192 (primed) at 4 WAP, n = 256 (non-primed)/n = 127 (primed) at 5 WAP, n = 64 (non-primed and BTH-treated)/n = 63 (primed) at 6 WAP, and n = 32 from 7 WAP for all treatments.

**Figure A6:**
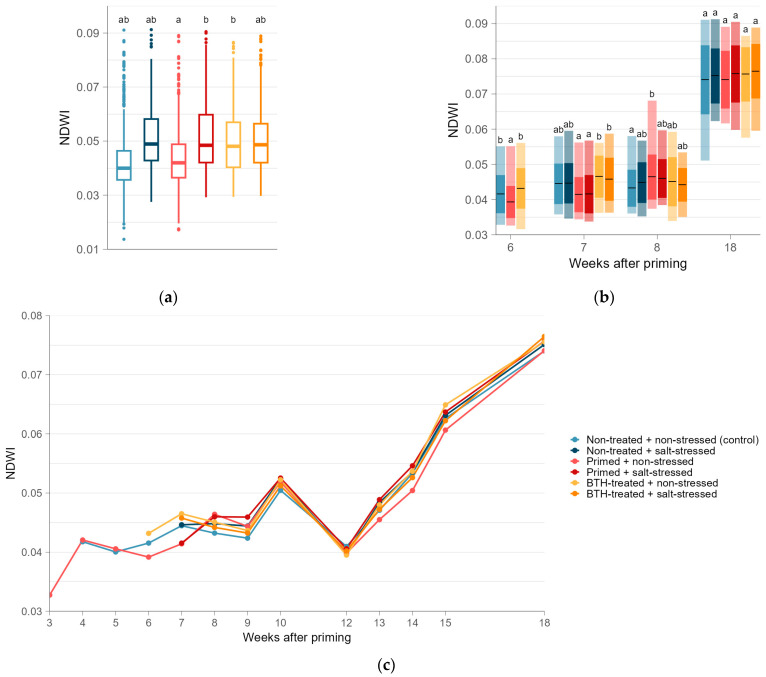
NDWI of young and mature tomato leaves, displayed by (**a**) boxplots over all dates, (**b**) mean (black horizonal line), standard deviation (intensely colored inner box), and maximum and minimum (lightly colored outer box) weeks after priming, and (**c**) trend lines representing means specific to each treatment week after priming, differentiated for the six treatments (from left to right): non-treated and non-stressed (control; light blue), non-treated and salt-stressed (dark blue), primed and non-stressed (light red), primed and salt-stressed (dark red), BTH-treated and non-stressed (yellow), and BTH-treated and salt-stressed (orange). The different letters indicate significant differences (ANOVA and EMMs post hoc; α = 0.05) between groups at the same week after priming. The sample size per treatment was as follows: n = 191 (non-primed)/n = 96 (primed) at 3 WAP, n = 384 (non-primed)/n = 192 (primed) at 4 WAP, n = 256 (non-primed)/n = 127 (primed) at 5 WAP, n = 64 (non-primed and BTH-treated)/n = 63 (primed) at 6 WAP, and n = 32 from 7 WAP for all treatments.

Additionally, the MFA revealed a negative correlation between the NDVI and NDWI (Figure 2a), both associated with vegetation health. Overall, the NDVI did not exhibit any effects (Figure A5a), whereas the primed and subsequently stressed group showed a higher NDWI than the non-stressed thermoprimed group (Figure A6a). The NDVI was initially decreased in primed plants until WAP 6 (Figure A5b,c). By WAP 7, the BTH-treated group had the lowest NDVI, while thermoprimed plants—with or without subsequent stress—displayed the highest NDVI. No treatment effects were found for the NDVI after 8 WAP until the end of the experiment (WAP 18). Then, the NDVI increased in the thermoprimed group compared to the control, but not compared to primed and subsequently stressed plants or between salt-stressed primed and non-primed plants. Conversely, the NDWI was initially increased in primed plants (Figure A6b,c). At WAP 6 and 7, thermoprimed plants showed a decreased NDWI compared to the non-primed control and BTH-treated plants. Thermoprimed plants exhibited a higher NDWI than the control.

**Figure A7:**
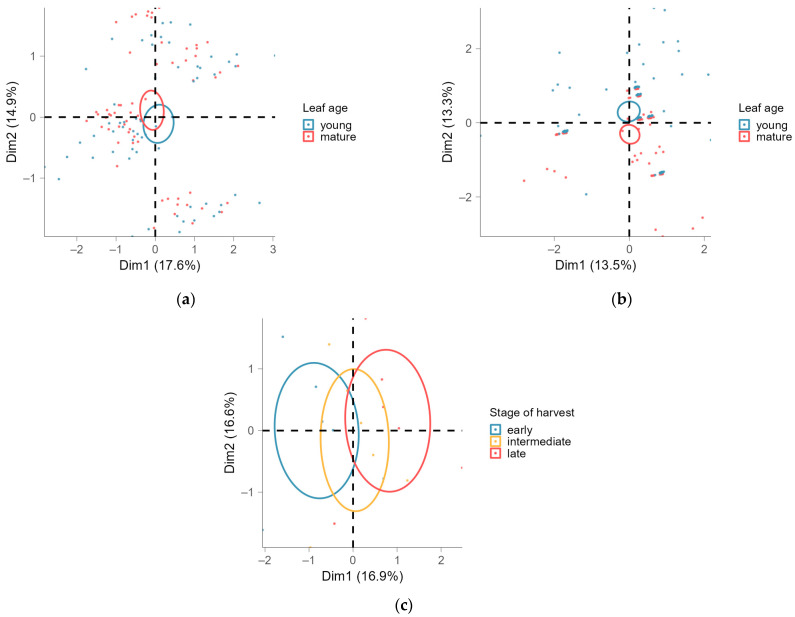
Multiple factor analysis (MFA) of standardized vegetation indices of tomato leaves (**a**), as well as primary and secondary compounds (TCC: total chlorophyll content; TCarC: total carotenoid content; TAC: total anthocyanin content; TPC: total phenolic content; FC: flavonoid content (expressed as CEs, catechin equivalents, or QEs, quercetin equivalents); Dualex chlorophyll (Chl) index; Dualex flavonol (Flav) index; Brix: dissolved sugar content; TA: titratable acidity) in leaves (**b**) and fruits (**c**) for group means (specified by treatment and either leaf age for leaf parameters and date or stage of harvest for fruit parameters), displayed as points grouped according to the young (light blue) or mature (light red) age of leaves (**a**,**b**) and the early (light blue), intermediate (yellow), or late (light red) stage of harvest (**c**) with confidence ellipses (β = 0.95). MFA was performed on the active variable, i.e., treatment, and the supplementary variables, i.e., date and leaf age for leaves and stage of harvest for fruits, as well as fruit color indices: a/b, color index, color difference (with) true red, and hue angle.

In addition to the individual effects of single VIs, we observed the separate clustering of the non-stressed control, thermoprimed, and BTH-treated groups in opposing quadrants (Figure 2b). Hence, these treatments induced different physiological reactions—at least concerning the assessed VIs. Salt stress predominantly affected the clustering of subsequently stressed groups apart from non-stressed groups. The leaf age did not influence the clustering of the VIs (Figure A7a).

### 2.2. Primary and Secondary Metabolites in Young and Mature Leaves

Overall, primed plants exhibited decreased TCC in young leaves compared to the non-primed control (−10%; Table A3). However, when subjected to subsequent salt stress, primed plants displayed the highest TCC. Similarly, the mature leaves of primed plants showed decreased TCC compared to non-primed plants (−10%). By the end of the experiment, the young and mature leaves of BTH-treated plants exhibited higher TCC than primed plants.

After thermopriming, the young leaves of primed and non-primed plants did not differ in their TCarC, while the BTH treatment led to decreased TCarC (Table A3). Subsequently stressed groups displayed the highest TCarC in young leaves, with only the primed, salt-stressed group showing a significant increase compared to the control. The TCarC in mature leaves was not significantly affected in the first few weeks after priming. However, after salt stress application, the mature leaves of primed (not stressed) plants exhibited the lowest TCarC, significantly less than the BTH-treated group.

Regarding the TAC in young leaves, no differences were found after thermopriming and subsequent stress (Table A3). However, the BTH treatment eventually led to decreased TAC compared to the control. In mature leaves, both thermopriming and BTH application caused a decrease in TAC. The subsequent salt stress did not affect the TAC or change this trend. By the end of the experiment, the mature leaves of primed and non-primed plants no longer differed in TAC, even in combination with subsequent stress. Overall, BTH resulted in decreased TAC in young and mature leaves (−13%).

The TPC was accumulated in the young leaves of thermoprimed plants (+5%), especially after subsequent stress (+12%), compared to the non-primed control, while the combination of BTH with subsequent stress resulted in the lowest TPC (Table A4). In mature leaves, primed plants displayed decreased TPC compared to non-primed plants after priming (−11%). BTH did not affect the phenols in mature leaves. By the end of the experiment, the TPC was increased in the young (+11%) and mature leaves (+15%) of primed, non-stressed plants compared to the control. In comparison, primed and subsequently stressed plants only showed an increased TPC trend.

Regarding FC selective for catechin, in young leaves, priming did not have an effect, while the FC selective for quercetin was temporarily decreased in primed plants. BTH also resulted in decreased FC_Catechin_ (Table A4). Mature leaves also indicated decreased FC after thermopriming. Similar to the observed effects in young leaves, subsequent stress was most significant for the accumulation of FC in all groups. By the end of the experiment, all subsequently salt-stressed treatments showed increased flavonoid content in young and mature leaves.

The Dualex flavonol index in young leaves was initially decreased in primed plants just after thermopriming (−25%; Table A5). However, thermoprimed plants exhibited a higher flavonol index in the following weeks until 4 WAP (up to +10%). Afterwards, there were no differences found between treatments, including after the BTH and salt application. By WAP 8, BTH-treated plants showed the lowest flavonol index (−10%). Moreover, in response to subsequent stress, non-primed plants had a higher flavonol index than primed plants. From WAP 9 to WAP 10, however, significantly decreased flavonol content was found in the control, BTH-treated, and primed, salt-stressed plants, whereas primed, non-stressed plants showed the highest flavonol index (up to +11%). Similar to WAP 8, BTH-treated plants displayed the lowest flavonol content at 12 WAP (−8% to control), although no differences were found between primed and non-primed and, respectively, salt-stressed and non-stressed plants. By WAP 14, we found a similar flavonol index in primed and subsequently stressed plants compared to the control, while primed and BTH-treated plants exhibited the highest flavonol index (+13% compared to the control). Then, at 15 WAP, the flavonol index was increased in primed plants compared to the control (+16%), while this effect was reversed again at 18 WAP (−15%). Overall, our treatments did not result in consistent effects on the flavonol indices of young leaves.

Regarding the flavonol index in mature leaves, there were no clear differences between treatments. However, at 4 WAP, the flavonol index in primed leaves was increased compared to non-primed leaves. In the following weeks (WAP 5 to 7), any effects of priming were not evident. During this period, we only observed decreased flavonol indices in BTH-treated plants (up to −6%). By WAP 9 and 10, the highest flavonol indices were exhibited in primed and subsequently salt-stressed plants (+15% more than control). Afterwards, the treatments no longer differed in terms of their flavonol indices in mature leaves.

**Figure 3:**
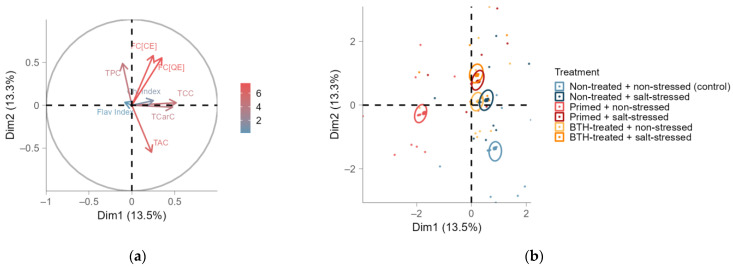
Multiple factor analysis (MFA) of standardized primary and secondary leaf compounds (TCC: total chlorophyll content; TCarC: total carotenoid content; TAC: total anthocyanin content; TPC: total phenolic content; FC: flavonoid content (expressed as CEs, catechin equivalents, or QEs, quercetin equivalents); Dualex chlorophyll (Chl) index; and Dualex flavonol (Flav) index) for group means (specified by treatment, leaf age, and date), displayed as points for the six treatments with confidence ellipses (β = 0.95; (**b**)), i.e., non-treated and non-stressed (control; light blue), non-treated and salt-stressed (dark blue), primed and non-stressed (light red), primed and salt-stressed (dark red), BTH-treated and non-stressed (yellow), and BTH-treated and salt-stressed (orange), or displayed as arrows for input parameters (**a**). MFA was performed on the active variable treatment as well as the supplementary variables: leaf age and date. The color gradient in (**a**) indicates the contributions of the variables to the dimensions (Dim).

The individual effects of all leaf parameters were comprehensively evaluated by MFA (Figure 3 and Figure A7b). Hereby, the analytically assessed FC values explained most of the variability in both dimensions, whereas the flavonol index, determined by the Dualex, contradictorily explained the least variability in the data (Figure 3a). These two groups were not correlated. Leaf pigments (TCC and TCarC) and FC had a positive correlation in between but not group-wise. Moreover, the accumulation of TPC and TAC in the leaves was not correlated with that of other leaf compounds. Hereby, the TPC and TAC were negatively correlated. We found an effect of the leaf age on the accumulation of primary and secondary compounds in the leaves (Figure A7b). However, these effects clearly underlaid those of the treatments (Figure 3b). The control, thermoprimed, and BTH-treated groups did cluster apart, while the BTH-treated group was associated with the subsequently salt-stressed groups. The latter were not differentiated in the MFA. Hence, thermopriming alone had an impact on leaf metabolites, but the later salt stress was predominant.

### 2.3. Secondary Metabolites in Fruits

**Figure 4:**
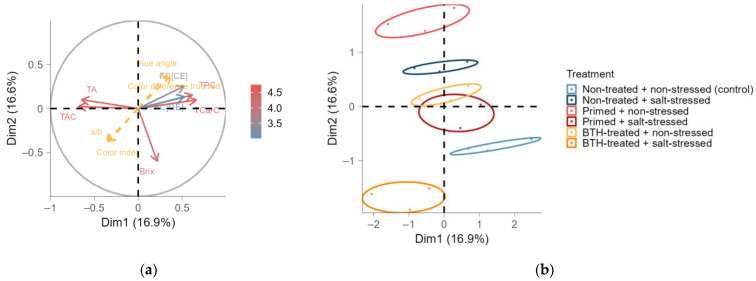
Multiple factor analysis (MFA) of standardized primary and secondary fruit compounds (TCC: total chlorophyll content; TCarC: total carotenoid content; TAC: total anthocyanin content; TPC: total phenolic content; FC: flavonoid content (expressed as CEs, catechin equivalents, or QEs, quercetin equivalents); Brix: dissolved sugar content; TA: titratable acidity) for group means (specified by treatment and stage of harvest) and the supplementary fruit color indices (a/b, color index, color difference (with) true red, and hue angle in yellow) displayed as points for the six treatments with confidence ellipses (β = 0.95; (**b**)), i.e., non-treated and non-stressed (control; light blue), non-treated and salt-stressed (dark blue), primed and non-stressed (light red), primed and salt-stressed (dark red), BTH-treated and non-stressed (yellow), and BTH-treated and salt-stressed (orange), or displayed as arrows for input parameters (**a**). MFA was performed on the active variable, i.e., treatment, as well as the supplementary variable, i.e., stage of harvest. The color gradient in (**a**) indicates the contributions of the variables to the dimensions (Dim).

The secondary metabolites in fruits at the early, intermediate, and late stages of harvest were assessed in addition to the vegetative growth parameters and leaf compounds. However, we did not observe any statistically significant effects on the Brix, titratable acidity, TCarC, TAC, TPC, and FC (Table A6 and Table A7). Therefore, we conducted an MFA to explore the underlying correlations and interactions among these fruit quality parameters (Figure 4 and Figure A7c). Despite the lack of differences in individual fruit parameters, we observed the separate clustering of the thermoprimed and control groups in opposing quadrants (Figure 4b). The salt-stressed groups were positioned opposite to the corresponding non-stressed groups with the same previous treatment. Notably, the TAC and titratable acidity explained most of the total variance in both dimensions (Figure 4a). Furthermore, the TAC, titratable acidity, TCarC, TPC, and FC showed positive correlations with each other, while both groups exhibited a negative correlation with each other. In contrast, Brix did not correlate with any parameter. The stages of harvest exhibited an overlap in their confidence ellipses (Figure A7c), suggesting differentiation in the inner fruit quality between the early and late stages of harvest.

## 3. Discussion

### 3.1. Thermopriming Triggered the Accumulation of Secondary Metabolites for Plant Defense

The trade-off in primary plant growth, benefitting the biosynthesis and accumulation of protective secondary metabolites in plant tissue, represents a well-known stress response, as comprehensively outlined by Martinez-Medina et al. [14]. Consistent with previous investigations by Körner et al. [12] and other studies [24,25,26,27], thermopriming initially delayed vegetative plant growth and development, manifesting in a reduced number of leaves (growth stage) and FM, but ultimately indicated accelerated plant development at a later developmental stage. Unlike the findings of Fan et al. [24], we did not observe any effects on dry matter. Thermoprimed plants generally displayed a decreased plant height and internode length, explicable physiologically by the influence of auxin thermomorphogenesis on hypocotyl elongation [28]. However, shortly following priming, the plant height and RGR showed an increase in response to the heat shock. In contrast to other experiments [12], tomato plants of the same cultivar recovered within a few weeks of thermopriming and did not sustain persistent growth impairments. Initially, thermopriming resulted in a decreased number of inflorescences and infructescences per plant, as previously reported [25,29], consequently affecting the fruit yield. Nonetheless, both primed and non-primed plants eventually realigned in their generative development. Primed plants likely recuperated from the heat shock without enduring adverse effects because they experienced heat stress only during the early developmental stage as seedlings, which might not have as severe an impact on the yield as during flowering or under prolonged mild heat [30]. The incidence of blossom-end rot remained unaffected by thermopriming in our study, neither positively nor negatively. Furthermore, no effects on the overall fruit quality were observed, consistent with findings from other studies [31,32].

Additionally, thermopriming led to overall decreased TCC in both young and mature leaves, which is attributable to high temperatures and is in line with reports by Fan et al. [24] and Berova et al. [33]. We also found a decrease in TCarC in leaves due to thermopriming. The TPC increased in both young and mature leaves by the end of the experiment, albeit experiencing an initial decline after thermopriming in mature leaves. Similar trends were observed for the TAC. Moreover, the flavonoid concentrations were reduced, primarily in mature leaves, while the flavonol index increased following thermopriming treatment, as indicated by the negative correlation between these groups in the MFA. Under stress conditions, flavonols are recognized as the principal subgroup of flavonoids that play a major role in various physiological responses [34]. Consequently, the pronounced differences between the epidermal flavonol indices non-invasively measured by the Dualex device and the FC of the entire leaves analyzed in the laboratory might be attributed to this. It is worth noting that the Dualex device measures the flavonols in the leaf epidermis by signal transmission through the leaf, while the FC was determined for the entire leaf, comprising both the epidermis and mesophyll parenchyma [35]. Flavonoids primarily accumulate in the vacuoles of mesophyll cells [36,37]. However, as epidermal flavonoids are dissolved in a much smaller tissue volume compared to the outer cell layers above the mesophyll tissue, changes in flavonoids may be more pronounced when analyzed using fluorescence measurement devices such as the Dualex [37].

Furthermore, our findings regarding the TCC were consistent with those of the spectral vegetation index NDVI, which corresponds to foliage greenness and, consequently, foliage health. Following an initial decrease, the water content, as determined by the NDVI, exhibited a temporary increase in the thermoprimed group. Conversely, the NDWI initially increased in primed plants and subsequently decreased, coinciding with the increase in the NDVI. Thus, while thermopriming induced water stress in the leaves even weeks after treatment, it did not adversely affect their health. In contrast to the TCarC, both CRI1 and CRI2 temporarily indicated the high accumulation of specific carotenoids in the tomato leaves.

### 3.2. Benzothiadiazole Impaired Plant Health and Growth without Increasing Stress Tolerance

We described the physiological effect of BTH on tomato plant growth and fruit development, particularly when subjected to subsequent salt stress. BTH, akin to salicylic acid in structure, has been successfully used in various studies to induce systemic plant resistance against biotic stress [15,16,17,18]. However, the literature regarding its comprehensive influence on plant development and yield performance under abiotic stress remains limited. Previous studies have reported that BTH can have adverse effects on both vegetative and generative plant growth, resulting in a decrease in the relative growth rate and impairing seed production across different plant species [38,39,40,41]. Godard et al. [42] have already elucidated the dosage-dependent reduction in growth attributed to BTH. Due to constraints in our greenhouse, our application of BTH was limited to drenching the root zone of each plant with a BTH solution. We opted for a concentration of BTH that had proven effective in combating nematodes without causing phytotoxicity to the treated plants, as demonstrated in a study by Molinari and Baser [43]. While alternative application methods of BTH might offer greater efficacy, they were not feasible within the scope of our experiment.

Dietrich et al. [38] observed initial growth depressions within the first week following BTH application, which were partly compensated for later, although the results varied due to unfavorable growing conditions. This suggests that the stressed plants may have been more profoundly affected by additional environmental stressors compared to the non-stressed plants. However, the tradeoff of BTH-induced defense against biotic and abiotic stress underscores the emphasized adverse effects on vegetative and generative growth. Yet, the question remains: do the physiological costs incurred in plant growth and development truly translate into long-term benefits for overall plant stress tolerance?

In our study, BTH resulted in even further delayed vegetative plant development—manifested by a decreased number of leaves and growth stage—compared to thermopriming, which induced growth deficits due to heat shock. BTH also induces a stress response in plants, consequently enhancing their stress tolerance. Although BTH temporarily impeded the tomato plants’ growth, it did not significant significantly affect the plant height, internode length, or relative growth rate. Sabir Tariq et al. [41] similarly demonstrated a negative impact of BTH on plant growth, characterized by stunted growth post-application. Overall, the total number of inflorescences and infructescences remained unaffected by BTH, although Azami-Sardooei et al. [40] noted reduced flower and fruit numbers in tomatoes following BTH treatment. In contrast to our findings, reports indicate that BTH delays fruit ripening [44] and can decrease fruit yields due to increased plant stress [41]. However, BTH led to a decline in fruit weight for early infructescences, particularly in combination with subsequent stress, while later trusses remained unaffected. Conversely, Louws et al. [45] generally observed no effects of BTH on the tomato fruit yield. In conclusion, BTH adversely impacted plant growth and development.

Regarding fruit disease resistance, previous studies have highlighted the beneficial impacts of BTH on fruit disease resistance [44,46,47]. However, our study, in contrast, revealed reduced resistance to plant diseases such as blossom-end rot, resulting in an increased loss of fruit yield midway through our experiment due to the higher number of non-marketable fruits. BTH-treated plants, when not under stress, were significantly more susceptible to blossom-end rot compared to other treatments, including the control. Interestingly, when subjected to subsequent stress, the BTH treatment showed no effect. Moreover, the food quality was generally unaffected by BTH, consistent with the findings of Cao and Jiang [48] in Yali pears but contrary to the findings of Cocetta et al. [49] in blueberry plants, who observed enhanced soluble content and diminished titratable acidity, as well as the increased accumulation of total polyphenols, flavonoids, and anthocyanins in fruits after BTH application. Similarly, Liu et al. [50] reported enhanced total phenolic compounds in peach fruits following postharvest treatment with BTH, aligning with the findings of Cao et al. [51] and Cao et al. [52], showing increased phenolic and anthocyanin content in strawberry fruits.

In our study, BTH led to a decrease in TCarC and TAC in the secondary metabolism of young leaves. While the mature leaves of BTH-treated plants were unaffected in their TCarC after subsequent stress, the TAC was further decreased compared to young leaves. By the end of the experiment, mature leaves showed higher TCarC compared to stressed non-treated plants. Compared to the control, the TPC did not significantly differ in the leaves, although stressed and previously BTH-treated plants exhibited lower TPC (trend) than in other treatments. BTH primarily resulted in decreased flavonoid content but temporarily led to the highest accumulation of flavonols in the leaves after stress compared to other groups. However, the response of the non-treated or primed groups to salt stress was similar to that of the BTH-treated groups. The influence of salt stress may have masked the positive effect of BTH on flavonol accumulation. In contrast, Sabir Tariq et al. [41] found no effect of BTH on chlorophylls a and b, carotenoids, lycopene, or the total phenolic content in tomato plants, while Hukkanen et al. [53] demonstrated the enhanced accumulation of phenolics in strawberry plants. The results for parameters related to the flavonoid content in leaves were inconsistent and contradictory throughout the cultivation period, particularly the non-invasively determined Dualex indices. At the end of the experiment, we observed an increased Dualex flavonol index in young leaves due to BTH, although it did not align with the FC.

By the end of the experiment, both the young and mature leaves of BTH-treated plants exhibited higher TCC. Additionally, the NDWI was higher in BTH-treated plants compared to the (non-stressed) thermoprimed group. Initially, the NDVI decreased after BTH application but later was not significantly different from the control. Overall, the greenness of the plants, indicative of their overall health, was enhanced by BTH. However, this observation did not correspond with the actual plant growth and fruit quality. Thus, BTH induced stress in plants without providing significant beneficial potential, as reported in other studies. Therefore, we do not recommend the use of BTH to enhance fruit disease resistance in tomato plants against abiotic stresses. Additionally, the partially long-lasting adverse effects of BTH on the plant physiology presented in this study should be considered in future experiments. Our study contributes to the existing knowledge on the use of chemical elicitors like BTH that can induce systemic plant resistance, although it did not serve as a positive control for thermopriming in our study.

### 3.3. Salinity Increased Flavonoid Accumulation, but This Occurred Too Late to Interact with Priming

In contrast to Zhang et al. [54], who reported the negative influence of salt stress in terms of a reduction in fruit number, size, and yield, we only found effects on fruit development caused by thermopriming. This was shown by the delayed fruit development of thermoprimed plants that were not under salt stress. Furthermore, the relatively late subsequent salt stress in this study did not significantly affect the growth stage, plant height, or RGR compared to other studies [8,55,56,57]. Regarding the internode length, the subsequent stress exacerbated the growth deficit of the previously thermoprimed plants, which can be explained by the inhibition of cell division and expansion in response to salt stress [58]. We also did not observe any interaction between priming or BTH with the subsequent stress—even when considering the final fruit yield or single fruit weight. Moreover, the subsequent salt stress did not have any effects on generative plant development (total number of inflorescences). Primed and subsequently stressed plants also showed reduced infructescences, but were not as severely affected as the only thermoprimed group. The other subsequently stressed groups did not differ significantly from the control group. Liu et al. [57] reported the decreased fresh matter of fruits after repeated treatment with a comparably mild salt concentration (50 mM NaCl solution). In our study, we treated the plants only once with a severe salt concentration (200 mM NaCl solution). Moreover, the salt stress was applied in a later development stage in our study. Older plants generally tolerate salt stress better than younger plants [7,59]. Salinity can increase the risk of fruit losses due to diseases such as blossom-end rot [60], but this was not the case in this experiment. The secondary metabolites, the Brix value, and the titratable acidity of the tomato fruits were also not noticeably affected. The influence of late salt stress on the accumulation of primary and secondary metabolites could also not be determined. However, in contrast to Rodrigues et al. [61], we found a trend towards increased TCC as well as significantly increased TCarC in the young leaves of previously thermoprimed and subsequently stressed plants, whereas salt stress did not affect these parameters in mature leaves. In addition, the flavonoid content in young and mature leaves in all groups after salt stress was significantly increased for FC_Quercetin_, but only in trend for FC_Catechin_. In contrast to the increase in the flavonol indices of young leaves due to thermopriming, we observed the opposite effect in response to the subsequent stress: primed and stressed plants temporarily showed a lower flavonol index than non-stressed, primed plants. In agreement with Martinez et al. [62], we found that flavonols accumulate under heat stress, whereas salt stress mainly leads to a downregulated response in a variety of flavonoids.

Our research indicates that the thermopriming of transplants can enhance the stress tolerance of tomato plants to a certain degree by promoting the accumulation of protective secondary metabolites in the leaves. However, this enhancement does not provide indefinite protection against abiotic stress. We suggest that the priming effect might require reactivation through controlled stress application to sustain increased plant defense over an extended period, especially if environmental stresses are not initially prominent post-transplanting in the greenhouse. This necessitates assessment, such as applying continuous stress conditions following transplant production during subsequent greenhouse cultivation. Through this approach, the efficacy of thermopriming can be practically determined. While plants can recover from single and temporary stress conditions, enduring continuous stresses pose greater challenges. Therefore, thermopriming could serve as a sustainable measure to protect plant production against climate-change-induced environmental uncertainties that threaten global food security.

## 4. Materials and Methods

### 4.1. Experimental and Priming Conditions

In 2022, a 20-week experiment was conducted at Geisenheim University (Geisenheim, Germany) from 14 March to 4 August to evaluate the plant growth and yield performance of truss tomato plants (*Solanum lycopersicum* L.) var. Adeleza (Enza Zaden Deutschland GmbH & Co. KG, Dannstadt-Schauernheim, Germany) that were treated either with thermopriming (two weeks after priming, WAP) or BTH (around 5 WAP), which was followed by a single instance of subsequent salt stress (around 6 WAP; Table 1 and Table 2). The priming was applied one week after sowing (WAS) for seven consecutive days in the form of a heat shock as ‘thermopriming’ under controlled conditions in climate chambers (Fitotron^®^ HGC 0714, Weiss Technik GmbH, Reiskirchen, Germany) at 40 °C for 90 min daily, according to Körner et al. [12]. For BTH application, 100 mL (*w*/*v*) aqueous BTH solution per plant (0.54 mg BTH mL^−1^ [43]) was administered as a positive control, alongside the thermopriming treatment. This approach aimed to stimulate plant resistance and compare its effectiveness with thermopriming. For the salt stress, a single dose of 100 mL 200 mM NaCl (EC: 20 dS m^−1^) was applied to each plant, while control plants received 100 mL distilled water instead of the BTH or salt solution.

Tomato seeds were sown in multipot ‘HerkuPak D 77′ plates (Herkuplast Kubern GmbH, Ering/Inn, Germany) with the peat substrate ‘Floradur A’ (Floragard Vertriebs-GmbH, Saterland, Germany). After priming at BBCH 12 [63], the transplants were potted in 10-cm-diameter pots filled with the peat substrate ‘Floradur B’ (Floragard Vertriebs-GmbH, Saterland, Germany). They were then placed in completely randomized blocks on tables in greenhouse chambers for further cultivation for 14 days (until BBCH 15), at temperatures of 22 °C during the day and 18 °C at night. Afterward, the transplants were transferred to a different greenhouse and transplanted in six rows of substrate ridges (Einheitserde SP Topf grob, PATZER ERDEN GmbH, Sinntal-Altengronau, Germany), with the two outer rows designated as borders without treatments. The pots were arranged according to a completely randomized block design for continued cultivation until 4 August. Each parcel/treatment (n = 6) and row/block (n = 4) included 12 plants. Following the BTH treatment, one plant on each end of all parcels was excluded from the measurements to prevent bias resulting from the surrounding and differently treated parcels. The sample size of the measurements decreased in the subsequent weeks from n = 14 plants per block to n = 12 at 21 April, n = 8 after the BTH treatment, and n = 4 after the salt stress. Plants were irrigated and fertigated according to Körner et al. [23].

### 4.2. Growth and Yield Parameters

In this study, we analyzed vegetative growth parameters, including the plant height, internode length, relative growth rate (calculated based on plant height), and number of leaves (principal growth stages defined by the BBCH scale [63]), as well as the number of inflorescences, number of infructescences, fruit yield, and single fruit weight, with regard to generative plant development. Additionally, we assessed fresh matter (FM) immediately after priming and at 2 WAP. At the end of the experiment, we recorded the overall above-ground dry matter and accumulated fresh matter. We thereby accounted for the FM of defoliated senescent leaves and evaluated the FM for plant leaves and stems separately. Due to the limited cultivation height in the greenhouse, plants were cut off on 15 June (13 WAS). Up until this point, the relative growth rate (RGR) was calculated weekly using the following equation [64]:

RGR = (ln H_2_ − ln H_1_)/(t_2_ − t_1_),

where H_1_ and H_2_ are the plant heights at times t_1_ and t_2_ (one week difference). RGR was not calculated at 1 WAP after potting and one week after transplanting into substrate ridges due to non-comparable differences in plant height.

During fruit development, trusses were reduced to six fruits per infructescence, following the recommendation of the cultivar’s breeder. Complete trusses were harvested twice weekly once all fruits per infructescence had visibly turned red. The weight of each truss and individual fruits was then assessed. Additionally, fruits from early (third truss), intermediate (fifth truss), and late (seventh truss) infructescences were measured using the CM-700d spectrophotometer (Konica Minolta Business Solutions Europe GmbH, Langenhagen, Germany) with gloss at three equatorial measurement spots on all six fruits to determine their average coloration at harvest. Color indices, including the hue angle (Hue), color index, color difference with true red, and a*/b*, were calculated based on the L*, a*, and b* values according to López Camelo and Gómez [65]. Afterward, one eighth of each fruit was frozen in liquid nitrogen as a mixed sample and stored at −80 °C.

### 4.3. Invasive Leaf and Fruit Analysis

Leaf samples were collected from both young (freshly formed, fully unfolded true leaves) and the oldest primary (mature) true leaves before and after the BTH and salt stress treatment, respectively, as well as at the end of the experiment. These samples were immediately frozen in liquid nitrogen and stored at −80 °C. Subsequently, fruit and leaf samples underwent colorimetric analysis according to Dörr et al. [66] to quantify the total chlorophyll content (TCC), total carotenoid content (TCarC), total anthocyanin content (TAC; expressed as cyanidin-3,5-O-diglucosid equivalents, CyEs), total phenolic content (TPC; expressed as gallic acid equivalents, GAEs), and flavonoid content (FC), which was selectively determined for (i) flavanols and flavones (FC_Quercetin_; expressed as quercetin equivalents, QEs) and (ii) rutin, luteolin, and catechin (FC_Catechin_; expressed as catechin equivalents, CEs) [67]. Three technical (undiluted) replicates were taken for each sample and averaged to reduce the potential technical bias introduced by the measurement device. Furthermore, the total titratable acidity (TA; expressed as CAEs, citric acid equivalents) and Brix were determined in accordance with Körner et al. [23].

### 4.4. Non-Invasive Leaf Measurements

Chlorophyll (Chl) and flavonoid (Flav) indices were non-invasively determined using a Dualex Scientific+™ device (Force-A, Orsay, France), which measures the leaf transmittance for the Chl index and fluorescence for the Flav index. For each leaf, three abaxial spots were measured—one on each side of the leaf and one at the tip. These technical replicates were then averaged to obtain the mean values.

The ASD FieldSpec^®^ 4 Standard-Res spectroradiometer (Malvern Panalytical Ltd., Spectris, London, UK), with a measurement range of 350 to 2500 nm, was used to compute the following vegetation indices (VIs) at a single central spot on the leaf tip of one young and mature leaf per plant, allowing the assessment of its stress and water status:

- Normalized Difference Vegetation Index (NDVI) [68];
- Red Edge Normalized Difference Vegetation Index (RENDVI) [69,70];
- Modified Red Edge Normalized Difference Vegetation Index (MRENDVI) [70,71];
- Vogelmann Red Edge Index 1 (VREI1) [72];
- Pigment Specific Simple Ratio a (PSSRa) [73];
- Pigment Specific Simple Ratio b (PSSRb) [73];
- Carotenoid Reflectance Index 1 (CRI1) [74];
- Carotenoid Reflectance Index 2 (CRI2) [74];
- Photochemical Reflectance Index (PRI) [75,76];
- Normalized Difference Water Index (NDWI) [77,78];
- Water (Band) Index (WBI) [79,80];
- Leaf Water Index (LWI) [81].

### 4.5. Data Analysis

For the statistical analysis of significant differences between treatments, ANOVA was conducted in R (version 4.2.2) using a linear mixed-effects model (α = 0.05; car package, version 3.1.1) in combination with the estimated marginal means post hoc (EMMs, α = 0.05, Tukey-adjusted; emmeans package, version 1.8.4.1) and the cld function (multcomp package, version 1.4.23) for pairwise comparisons (α = 0.05) to identify individual treatment effects. The lmer models (lmerTest package, version 3.1.3) were specified based on random variables such as the leaf age (for leaf parameters) or stage of harvest and fruit coloration as covariates (for fruit parameters), alongside overall random effects accounting for the completely randomized block design and repeated measurements. The best model for each fruit parameter was determined by comparing models with different color indices using the performance package (version 0.10.2). Moreover, multiple factor analysis (MFA) was carried out using the factoextra package (version 1.0.7). Prior to MFA, the Vis and Dualex indices, as well as the leaf and fruit compounds, underwent outlier removal based on the interquartile range criterion. Plots were created using the ggplot2 package (version 3.4.1).

### 4.6. Multiple Factor Analysis

MFA was separately conducted for the hyperspectral leaf VIs, as well as the primary and secondary plant metabolites in both the leaf and fruit, with the treatment serving as the active variable. Supplementary variables such as the leaf age and date of measurement were incorporated for leaf indices and compounds, while fruit coloration (Hue, color index, color difference with true red, and a*/b*) and the stage of harvest (early, intermediate, and late) were added for fruit parameters. Regarding the fruit analysis, MFA was applied to the colorimetrically measured compounds (TCarC, TAC, TPC, and FCs), as well as Brix and TA, which served as quantitative input variables. For leaves, two separate MFAs were performed: one encompassing all primary and secondary leaf compounds and another for the hyperspectral VIs as input variables.

## Figures and Tables

**Table 1:** Summary of experimental settings.

Duration	20 weeks
Period	March–August 2022
Timing of thermopriming (week after sowing)	2nd
Timing of BTH application (days after priming)	37
Timing of salt stress (days after priming)	44
Number of treatments	6
Total number of blocks (including border)	6
Number of blocks with experimental plants	4
Total number of plants (including border) per block	14
Number of experimental plants per block	12/8/4
Total number of experimental plants per treatment	48/32/16
Number of harvested trusses per experimental plant	3 (3rd/5th/7th truss per plant)

**Table 2:** Timeline with experimental treatments.

Treatment	Weeks after Sowing																					
	1	2	3	4	5	6	7		8		9	10	11	12	13	14	15	16	17	18	19	20
C–C																						
C–S																						
B–C																						
B–S																						
P–C																						
P–S																						

Notes: Seeding and 7-day thermopriming (light red) in climate chambers, one single BTH application (yellow), and one single salt stress application (blue) during a 20-week greenhouse cultivation (light grey), as well as four dates for leaf sampling (dark red vertical lines) and 7-week-long duration of fruit sampling (dark grey) specific to each of the six treatments over the whole experimental duration in weeks after sowing. Treatments: B represents the BTH treatment, P represents thermopriming, C represents control conditions (non-primed or non-stressed groups), and S represents salt stress (e.g., C–C: control conditions (non-primed) instead of priming or BTH treatment and non-stressed (C) at the time of the salt stress).

## Data Availability

The data presented in this study are available on request.

## Appendix A

**Table A1:** Fresh matter (FM) and dry matter (DM) of tomato plants by the end of the experiment, displayed as the mean and standard deviation differentiated for the six treatments.

Treatment	FM Leaf	FM Stem	FM Total ^1^	DM Leaf	DM Stem	DM Total ^2^	n
	[g]						
Non-treated + non-stressed	722 ± 143 ^a^	839 ± 91 ^a^	2581 ± 282 ^a^	134 ± 21 ^a^	167 ± 20 ^a^	300 ± 40 ^a^	16
Non-treated + salt-stressed	749 ± 128 ^a^	822 ± 110 ^a^	2558 ± 272 ^a^	134 ± 21 ^a^	172 ± 25 ^a^	307 ± 44 ^a^	16
Primed + non-stressed	714 ± 117 ^a^	815 ± 93 ^a^	2514 ± 256 ^a^	126 ± 19 ^a^	171 ± 15 ^a^	297 ± 33 ^a^	16
Primed + salt-stressed	709 ± 113 ^a^	796 ± 92 ^a^	2537 ± 235 ^a^	134 ± 21 ^a^	169 ± 19 ^a^	303 ± 39 ^a^	16
BTH ^3^-treated + non-stressed	667 ± 113 ^a^	796 ± 84 ^a^	2466 ± 195 ^a^	124 ± 16 ^a^	165 ± 17 ^a^	290 ± 32 ^a^	16
BTH-treated + salt-stressed	703 ± 183 ^a^	742 ± 133 ^a^	2414 ± 287 ^a^	124 ± 24 ^a^	154 ± 27 ^a^	278 ± 49 ^a^	16

^1^ FM total: final FM including defoliated leaves; ^2^ DM total: final DM including defoliated leaves; ^3^ BTH: benzothiadiazole. Notes: the different letters indicate significant differences (ANOVA and EMMs post hoc; α = 0.05) between groups in the same week after priming for the corresponding parameter. n corresponds to the sample size of the previous parameters.

**Table A2:** Accumulated fruit yield, displayed as the mean and standard deviation differentiated for the six treatments.

Treatment	WAP ^1^	Accumulated Fruit Yield	n	Treat.	WAP	Accumulated Fruit Yield	n
		[g]				[g]	
Non-treated + non-stressed (control)	12	693.7 ± 274.2 ^a^	16	Primed + salt-stressed	12	677.0 ± 186.8 ^a^	16
	13	2116.2 ± 588.2 ^a^	32		13	2036.7 ± 342.3 ^a^	32
	14	2530.8 ± 649.6 ^a^	15		14	2300.9 ± 372.9 ^a^	16
	15	2685.6 ± 946.2 ^a^	17		15	2936.3 ± 481.3 ^a^	16
	16	3348.1 ± 767.2 ^a^	16		16	3522.8 ± 561.8 ^a^	16
	17	4362.7 ± 642.5 ^ab^	16		17	4548.0 ± 593.5 ^ab^	16
Non-treated + salt-stressed	12	573.4 ± 81.0 ^a^	16	BTH ^2^-treated + non-stressed	12	797.4 ± 278.4 ^a^	16
	13	2179.2 ± 397.4 ^a^	32		13	1880.8 ± 445.6 ^a^	32
	14	2647.8 ± 414.9 ^a^	16		14	2233.3 ± 486.4 ^a^	16
	15	3135.5 ± 355.8 ^a^	16		15	2786.5 ± 637.5 ^a^	16
	16	3702.6 ± 357.8 ^a^	16		16	3218.5 ± 618.3 ^a^	16
	17	4727.1 ± 307.7 ^b^	16		17	4072.9 ± 728.0 ^a^	16
Primed + non-stressed	12	687.9 ± 210.4 ^a^	16	BTH-treated + salt-stressed	12	761.7 ± 264.7 ^a^	16
	13	1891.6 ± 365.1 ^a^	32		13	2098.3 ± 558.3 ^a^	32
	14	2183.0 ± 452.6 ^a^	16		14	2457.4 ± 464.5 ^a^	16
	15	2849.4 ± 445.2 ^a^	16		15	2991.9 ± 476.1 ^a^	16
	16	3361.3 ± 563.0 ^a^	16		16	3527.5 ± 518.5 ^a^	16
	17	4467.5 ± 550.9 ^ab^	16		17	4451.1 ± 584.9 ^ab^	16

^1^ WAP: week after priming; ^2^ BTH: benzothiadiazole. Notes: the different letters indicate significant differences (ANOVA and EMMs post hoc; α = 0.05) between groups in the same week after priming for the corresponding parameter. n corresponds to the sample size of the previous parameters.

**Table A3:** Leaf compounds, displayed as the mean and standard deviation in young and mature tomato leaves, differentiated for the six treatments (part 1 of 2).

Treatment	WAP ^1^	Leaf Age	TCC ^2^	TCarC ^3^	TAC ^4^	n
			[µg mg^−1^ DM^−1^]		[µg CyEs mg^−1^ DM^−1^]	
Non-treated + non-stressed (control)	overall	young	3.1 ± 0.8 ^b^	1.7 ± 0.5 ^b^	1.9 ± 0.5 ^b^	128
	5		3.6 ± 0.4 ^a^	2.1 ± 0.3 ^a^	2.0 ± 0.6 ^a^	64
	6		3.1 ± 0.4 ^a^	1.5 ± 0.3 ^b^	1.8 ± 0.4 ^a^	32
	7		2.9 ± 0.5 ^a^	1.4 ± 0.3 ^ab^	1.6 ± 0.5 ^a^	16
	18		1.3 ± 0.2 ^ab^	1.2 ± 0.3 ^a^	1.9 ± 0.4 ^b^	16
	overall	mature	3.3 ± 0.6 ^b^	1.8 ± 0.5 ^b^	2.4 ± 0.6 ^c^	128
	5		3.3 ± 0.6 ^b^	2.0 ± 0.5 ^a^	2.1 ± 0.5 ^b^	64
	6		3.5 ± 0.5 ^a^	1.7 ± 0.4 ^a^	2.6 ± 0.6 ^b^	32
	7		3.2 ± 0.6 ^bc^	1.3 ± 0.5 ^ab^	2.6 ± 0.6 ^b^	16
	18		2.7 ± 0.3 ^ab^	1.8 ± 0.6 ^ab^	3.0 ± 0.4 ^c^	16
Non-treated + salt-stressed	overall	young	2.0 ± 0.9 ^b^	1.3 ± 0.4 ^ab^	1.7 ± 0.5 ^ab^	30
	7		2.9 ± 0.3 ^a^	1.5 ± 0.4 ^abc^	1.6 ± 0.6 ^a^	14
	18		1.2 ± 0.3 ^ab^	1.1 ± 0.3 ^a^	1.7 ± 0.4 ^ab^	16
	overall	mature	2.7 ± 0.7 ^b^	1.4 ± 0.5 ^ab^	2.7 ± 0.5 ^b^	30
	7		3.0 ± 0.8 ^bc^	1.4 ± 0.6 ^ab^	2.5 ± 0.6 ^b^	14
	18		2.4 ± 0.4 ^ab^	1.4 ± 0.3 ^a^	2.8 ± 0.4 ^c^	16
Primed + non-stressed	overall	young	2.8 ± 0.9 ^a^	1.5 ± 0.5 ^a^	1.7 ± 0.5 ^ab^	96
	5		3.5 ± 0.5 ^a^	2.0 ± 0.3 ^a^	1.8 ± 0.6 ^a^	32
	6		3.0 ± 0.4 ^a^	1.4 ± 0.2 ^ab^	1.8 ± 0.4 ^a^	32
	7		2.6 ± 0.4 ^a^	1.2 ± 0.3 ^a^	1.5 ± 0.6 ^a^	16
	18		1.0 ± 0.2 ^a^	1.1 ± 0.3 ^a^	1.6 ± 0.6 ^ab^	16
	overall	mature	2.9 ± 0.7 ^a^	1.6 ± 0.4 ^a^	2.1 ± 0.5 ^ab^	96
	5		3.0 ± 0.6 ^a^	1.8 ± 0.4 ^a^	1.8 ± 0.4 ^a^	32
	6		3.2 ± 0.7 ^a^	1.6 ± 0.4 ^a^	2.2 ± 0.6 ^a^	32
	7		2.6 ± 0.5 ^a^	1.2 ± 0.4 ^a^	1.9 ± 0.3 ^a^	16
	18		2.2 ± 0.4 ^a^	1.5 ± 0.4 ^ab^	2.6 ± 0.4 ^bc^	16
Primed + salt-stressed	overall	young	2.1 ± 0.9 ^b^	1.4 ± 0.4 ^b^	1.6 ± 0.4 ^ab^	32
	7		2.9 ± 0.3 ^a^	1.7 ± 0.3 ^c^	1.7 ± 0.5 ^a^	16
	18		1.3 ± 0.2 ^ab^	1.2 ± 0.3 ^a^	1.6 ± 0.4 ^ab^	16
	overall	mature	2.7 ± 0.6 ^b^	1.6 ± 0.6 ^ab^	2.3 ± 0.5 ^a^	31
	7		2.8 ± 0.6 ^ab^	1.5 ± 0.5 ^ab^	1.9 ± 0.3 ^a^	15
	18		2.5 ± 0.6 ^ab^	1.7 ± 0.6 ^ab^	2.7 ± 0.4 ^bc^	16
BTH ^5^-treated + non-stressed	overall	young	2.5 ± 0.8 ^ab^	1.3 ± 0.3 ^ab^	1.6 ± 0.4 ^a^	64
	6		3.0 ± 0.4 ^a^	1.3 ± 0.2 ^a^	1.8 ± 0.5 ^a^	32
	7		2.8 ± 0.4 ^a^	1.4 ± 0.4 ^ab^	1.4 ± 0.2 ^a^	16
	18		1.3 ± 0.2 ^b^	1.3 ± 0.4 ^a^	1.3 ± 0.3 ^a^	16
	overall	mature	3.2 ± 0.7 ^b^	1.7 ± 0.4 ^ab^	2.2 ± 0.4 ^a^	63
	6		3.3 ± 0.7 ^a^	1.6 ± 0.4 ^a^	2.2 ± 0.4 ^a^	32
	7		3.5 ± 0.7 ^c^	1.6 ± 0.5 ^b^	2.1 ± 0.3 ^a^	15
	18		2.7 ± 0.5 ^b^	1.8 ± 0.4 ^ab^	2.4 ± 0.3 ^ab^	16
BTH-treated + salt-stressed	overall	young	2.0 ± 1.0 ^ab^	1.3 ± 0.5 ^ab^	1.5 ± 0.4 ^ab^	32
	7		2.9 ± 0.6 ^a^	1.5 ± 0.6 ^bc^	1.6 ± 0.3 ^a^	16
	18		1.1 ± 0.3 ^ab^	1.1 ± 0.4 ^a^	1.4 ± 0.4 ^a^	16
	overall	mature	2.9 ± 0.6 ^b^	1.7 ± 0.7 ^b^	2.1 ± 0.3 ^a^	32
	7		3.1 ± 0.3 ^bc^	1.4 ± 0.4 ^ab^	1.9 ± 0.3 ^a^	16
	18		2.7 ± 0.7 ^ab^	2.0 ± 0.9 ^b^	2.3 ± 0.3 ^a^	16

^1^ WAP: week after priming; ^2^ TCC: total chlorophyll content; ^3^ TCarC: total carotenoid content; ^4^ TAC: total anthocyanin content (expressed as CyEs, cyanidin-3,5-O-diglucosid equivalents); ^5^ BTH: benzothiadiazole. Notes: the different letters indicate significant differences (ANOVA and EMMs post hoc; α = 0.05) between groups in the same week after priming for the corresponding parameter. n corresponds to the sample size of the previous parameters.

**Table A4:** Leaf compounds, displayed as the mean and standard deviation in young and mature tomato leaves, differentiated for the six treatments (part 2 of 2).

Treatment	WAP ^1^	Leaf Age	TPC ^2^	FC_Catechin_ ^3^	FC_Quercetin_ ^3^	n
			[µg GAEs mg^−1^ DM^−1^]	[µg CEs mg^−1^ DM^−1^]	[µg QEs mg^−1^ DM^−1^]	
Non-treated + non-stressed (control)	overall	young	14.8 ± 1.8 ^a^	21.8 ± 4.6 ^a^	14.1 ± 2.2 ^a^	128
	5		13.6 ± 1.2 ^a^	24.1 ± 2.7 ^a^	14.8 ± 0.8 ^a^	64
	6		15.7 ± 1.9 ^a^	21.1 ± 2.9 ^b^	14.6 ± 1.7 ^b^	32
	7		16.3 ± 1.3 ^a^	24.6 ± 3.0 ^a^	14.8 ± 1.9 ^a^	16
	18		15.1 ± 1.6 ^a^	13.0 ± 1.4 ^ab^	10.2 ± 2.6 ^a^	16
	overall	mature	7.6 ± 2.1 ^a^	14.2 ± 2.3 ^ab^	11.9 ± 1.6 ^b^	128
	5		6.7 ± 0.9 ^b^	14.4 ± 2.4 ^b^	12.0 ± 1.0 ^b^	64
	6		7.0 ± 0.7 ^b^	14.4 ± 2.2 ^b^	12.8 ± 1.2 ^b^	32
	7		7.3 ± 0.6 ^a^	13.1 ± 1.5 ^bc^	11.5 ± 1.7 ^b^	16
	18		12.5 ± 1.5 ^a^	13.7 ± 2.0 ^ab^	10.3 ± 2.4 ^a^	16
Non-treated + salt-stressed	overall	young	16.4 ± 2.0 ^a^	19.6 ± 5.6 ^a^	13.6 ± 3.6 ^a^	30
	7		16.9 ± 1.6 ^ab^	24.7 ± 2.2 ^a^	16.2 ± 1.6 ^bc^	14
	18		16.0 ± 2.2 ^ab^	15.2 ± 3.4 ^bc^	11.3 ± 3.4 ^bc^	16
	overall	mature	10.1 ± 2.8 ^a^	14.1 ± 2.0 ^b^	11.9 ± 2.6 ^b^	30
	7		7.4 ± 0.4 ^a^	13.3 ± 1.7 ^bc^	12.6 ± 1.5 ^c^	14
	18		12.4 ± 1.6 ^a^	14.8 ± 2.0 ^ab^	11.4 ± 3.2 ^ab^	16
Primed + non-stressed	overall	young	15.4 ± 2.1 ^a^	21.1 ± 4.9 ^a^	13.7 ± 2.3 ^a^	96
	5		13.4 ± 1.6 ^a^	23.4 ± 3.3 ^a^	14.6 ± 1.0 ^a^	32
	6		15.6 ± 1.5 ^a^	20.4 ± 2.3 ^ab^	13.9 ± 1.3 ^a^	32
	7		17.3 ± 1.5 ^ab^	26.1 ± 3.7 ^a^	15.2 ± 2.1 ^ab^	16
	18		16.8 ± 1.4 ^b^	13.5 ± 1.3 ^abc^	10.3 ± 2.8 ^ab^	16
	overall	mature	7.8 ± 3.0 ^a^	13.2 ± 2.2 ^a^	11.3 ± 1.8 ^a^	96
	5		6.3 ± 0.7 ^a^	13.5 ± 2.1 ^a^	11.3 ± 1.1 ^a^	32
	6		6.5 ± 0.5 ^a^	13.1 ± 2.7 ^a^	12.0 ± 1.3 ^a^	32
	7		7.2 ± 0.6 ^a^	11.5 ± 1.0 ^a^	10.2 ± 1.8 ^a^	16
	18		14.4 ± 1.1 ^b^	14.5 ± 1.1 ^ab^	10.9 ± 2.8 ^a^	16
Primed + salt-stressed	overall	young	17.1 ± 2.1 ^a^	20.7 ± 5.9 ^a^	14.0 ± 3.5 ^a^	32
	7		18.2 ± 0.9 ^b^	25.8 ± 1.8 ^a^	16.7 ± 1.2 ^c^	16
	18		16.0 ± 2.3 ^ab^	15.6 ± 3.7 ^c^	11.4 ± 3.1 ^c^	16
	overall	mature	10.3 ± 3.3 ^a^	13.9 ± 2.8 ^ab^	12.1 ± 2.9 ^b^	31
	7		7.1 ± 0.6 ^a^	12.1 ± 1.3 ^ab^	11.9 ± 1.4 ^bc^	15
	18		13.4 ± 1.3 ^ab^	15.7 ± 2.6 ^b^	12.4 ± 3.9 ^b^	16
BTH ^4^-treated + non-stressed	overall	young	15.8 ± 1.6 ^a^	19.4 ± 5.0 ^a^	13.3 ± 2.7 ^a^	64
	6		15.6 ± 1.4 ^a^	20.0 ± 2.1 ^a^	14.1 ± 1.4 ^ab^	32
	7		16.9 ± 1.5 ^ab^	25.0 ± 3.0 ^a^	15.1 ± 2.1 ^ab^	16
	18		15.1 ± 1.5 ^a^	12.4 ± 1.4 ^a^	9.9 ± 2.2 ^a^	16
	overall	mature	8.4 ± 2.7 ^a^	13.8 ± 2.4 ^ab^	11.9 ± 1.9 ^ab^	63
	6		6.8 ± 0.4 ^ab^	13.9 ± 2.7 ^ab^	12.3 ± 1.3 ^ab^	32
	7		7.3 ± 0.4 ^a^	13.9 ± 1.8 ^c^	11.8 ± 1.8 ^bc^	15
	18		12.8 ± 1.4 ^a^	13.5 ± 2.5 ^a^	11.1 ± 2.5 ^a^	16
BTH-treated + salt-stressed	overall	young	16.2 ± 1.9 ^a^	19.7 ± 6.1 ^a^	13.6 ± 3.7 ^a^	32
	7		17.4 ± 1.5 ^ab^	25.0 ± 1.7 ^a^	16.3 ± 1.4 ^c^	16
	18		15.0 ± 1.4 ^a^	14.3 ± 3.4 ^abc^	10.9 ± 3.3 ^abc^	16
	overall	mature	10.0 ± 3.1 ^a^	13.9 ± 2.6 ^b^	11.7 ± 2.6 ^b^	32
	7		7.3 ± 0.5 ^a^	12.7 ± 1.4 ^abc^	12.2 ± 1.0 ^bc^	16
	18		12.8 ± 1.7 ^a^	15.2 ± 3.0 ^ab^	11.2 ± 3.6 ^ab^	16

^1^ WAP: week after priming; ^2^ TPC: total phenolic content (expressed as GAEs, gallic acid equivalents); ^3^ FC: flavonoid content (expressed as CEs, catechin equivalents, or QEs, quercetin equivalents); ^4^ BTH: benzothiadiazole. Notes: the different letters indicate significant differences (ANOVA and EMMs post hoc; α = 0.05) between groups in the same week after priming for the corresponding parameter. n corresponds to the sample size of the previous parameters.

**Table A5:** Dualex flavonol (Flav) index, displayed as the mean and standard deviation in young and mature tomato leaves, differentiated for the six treatments (part 2 of 2).

Treatment	WAP ^1^	Leaf Age	Dualex Flav Index	n	Treat.	WAP	Leaf Age	Dualex Flav Index		n
Non-treated + non-stressed (control)	overall	young	0.58 ± 0.17 ^a^	3430	Primed + non-stressed	overall	young	0.63 ± 0.20 ^a^		2046
	0		0.50 ± 0.09 ^b^	270		0		0.37 ± 0.06 ^a^		131
	1		0.47 ± 0.07 ^a^	672		1		0.47 ± 0.07 ^a^		336
	2		0.62 ± 0.12 ^a^	670		2		0.68 ± 0.12 ^b^		335
	3		0.48 ± 0.09 ^a^	572		3		0.52 ± 0.11 ^b^		285
	4		0.55 ± 0.11 ^a^	575		4		0.57 ± 0.12 ^a^		288
	5		0.68 ± 0.12 ^a^	192		5		0.68 ± 0.12 ^a^		192
	6		0.75 ± 0.13 ^ab^	48		6		0.80 ± 0.13 ^b^		48
	7		0.81 ± 0.12 ^a^	48		7		0.81 ± 0.14 ^a^		48
	8		0.83 ± 0.12 ^bc^	48		8		0.79 ± 0.13 ^abc^		48
	9		0.88 ± 0.14 ^a^	48		9		0.98 ± 0.10 ^b^		47
	10		0.79 ± 0.07 ^ab^	47		10		0.84 ± 0.13 ^b^		48
	12		0.78 ± 0.08 ^b^	48		12		0.76 ± 0.12 ^ab^		48
	13		0.91 ± 0.13 ^a^	48		13		0.85 ± 0.16 ^a^		48
	14		0.88 ± 0.15 ^a^	48		14		0.99 ± 0.18 ^b^		48
	15		0.87 ± 0.17 ^a^	48		15		1.01 ± 0.21 ^b^		48
	18		0.99 ± 0.25 ^b^	48		18		0.84 ± 0.15 ^a^		48
	overall	mature	0.47 ± 0.10 ^a^	1247		overall	mature	0.46 ± 0.11 ^a^		959
	4		0.51 ± 0.08 ^a^	576		4		0.52 ± 0.09 ^b^		288
	5		0.45 ± 0.05 ^b^	192		5		0.45 ± 0.05 ^b^		191
	6		0.45 ± 0.07 ^b^	48		6		0.44 ± 0.04 ^ab^		48
	7		0.43 ± 0.06 ^ab^	48		7		0.43 ± 0.05 ^ab^		48
	8		0.34 ± 0.06 ^a^	48		8		0.35 ± 0.07 ^a^		48
	9		0.31 ± 0.05 ^a^	48		9		0.34 ± 0.08 ^ab^		48
	10		0.32 ± 0.05 ^a^	48		10		0.36 ± 0.09 ^ab^		48
	12		0.34 ± 0.04 ^a^	48		12		0.36 ± 0.07 ^a^		48
	13		0.50 ± 0.09 ^a^	47		13		0.50 ± 0.10 ^a^		48
	14		0.48 ± 0.11 ^a^	48		14		0.46 ± 0.09 ^a^		48
	15		0.51 ± 0.08 ^a^	48		15		0.53 ± 0.08 ^a^		48
	18		0.62 ± 0.13 ^a^	48		18		0.59 ± 0.14 ^a^		48
Non-treated + salt-stressed	overall	young	0.86 ± 0.16 ^a^	479	Primed + salt-stressed	overall	young	0.83 ± 0.14 ^a^		479
	6		0.72 ± 0.11 ^a^	48		6		0.74 ± 0.11 ^ab^		48
	7		0.79 ± 0.11 ^a^	48		7		0.79 ± 0.11 ^a^		48
	8		0.85 ± 0.14 ^c^	48		8		0.76 ± 0.13 ^ab^		48
	9		0.94 ± 0.11 ^ab^	47		9		0.90 ± 0.11 ^a^		48
	10		0.81 ± 0.13 ^ab^	48		10		0.80 ± 0.12 ^ab^		48
	12		0.77 ± 0.09 ^b^	48		12		0.74 ± 0.12 ^ab^		48
	13		0.90 ± 0.13 ^a^	48		13		0.85 ± 0.13 ^a^		48
	14		0.95 ± 0.16 ^ab^	48		14		0.87 ± 0.10 ^a^		48
	15		0.93 ± 0.18 ^ab^	48		15		0.91 ± 0.13 ^a^		48
	18		0.97 ± 0.17 ^b^	48		18		0.89 ± 0.20 ^ab^		47
Non-treated + salt-stressed	overall	mature	0.44 ± 0.14 ^a^	480		overall	mature	0.44 ± 0.12 ^a^		480
	6		0.45 ± 0.04 ^b^	48		6		0.44 ± 0.05 ^ab^		48
	7		0.44 ± 0.06 ^b^	48		7		0.43 ± 0.05 ^ab^		48
	8		0.34 ± 0.09 ^a^	48		8		0.37 ± 0.09 ^a^		48
	9		0.32 ± 0.08 ^ab^	48		9		0.35 ± 0.08 ^b^		48
	10		0.33 ± 0.08 ^ab^	48		10		0.36 ± 0.09 ^b^		48
	12		0.35 ± 0.06 ^a^	48		12		0.37 ± 0.08 ^a^		48
	13		0.50 ± 0.12 ^a^	48		13		0.51 ± 0.11 ^a^		48
	14		0.46 ± 0.13 ^a^	48		14		0.47 ± 0.12 ^a^		48
	15		0.53 ± 0.15 ^a^	48		15		0.50 ± 0.11 ^a^		48
	18		0.65 ± 0.11 ^a^	48		18		0.64 ± 0.09 ^a^		48
BTH ^2^-treated + non-stressed	overall	young	0.80 ± 0.17 ^a^	672	BTH-treated + salt-stressed	overall	young	0.84 ± 0.15 ^a^		480
	5		0.70 ± 0.12 ^a^	192						
	6		0.75 ± 0.12 ^ab^	48		6		0.79 ± 0.13 ^b^		48
	7		0.80 ± 0.12 ^a^	48		7		0.76 ± 0.12 ^a^		48
	8		0.75 ± 0.12 ^a^	48		8		0.78 ± 0.12 ^abc^		48
	9		0.90 ± 0.15 ^a^	48		9		0.92 ± 0.12 ^ab^		48
	10		0.75 ± 0.10 ^a^	48		10		0.78 ± 0.14 ^ab^		48
	12		0.71 ± 0.11 ^a^	48		12		0.73 ± 0.10 ^ab^		48
	13		0.88 ± 0.13 ^a^	48		13		0.90 ± 0.09 ^a^		48
	14		0.99 ± 0.19 ^b^	48		14		0.94 ± 0.17 ^ab^		48
	15		0.85 ± 0.11 ^a^	48		15		0.93 ± 0.13 ^ab^		48
	18		0.97 ± 0.24 ^b^	48		18		0.85 ± 0.14 ^a^		48
	overall	mature	0.43 ± 0.10 ^a^	671		overall	mature	0.42 ± 0.12 ^a^		479
	5		0.43 ± 0.05 ^a^	192						
	6		0.42 ± 0.05 ^a^	48		6		0.42 ± 0.05 ^a^		48
	7		0.40 ± 0.06 ^a^	48		7		0.41 ± 0.05 ^a^		48
	8		0.35 ± 0.06 ^a^	48		8		0.34 ± 0.07 ^a^		48
	9		0.32 ± 0.05 ^ab^	48		9		0.32 ± 0.06 ^ab^		48
	10		0.35 ± 0.06 ^ab^	48		10		0.34 ± 0.06 ^ab^		48
	12		0.36 ± 0.05 ^a^	48		12		0.36 ± 0.06 ^a^		48
	13		0.52 ± 0.08 ^a^	47		13		0.48 ± 0.11 ^a^		47
	14		0.49 ± 0.09 ^a^	48		14		0.46 ± 0.11 ^a^		48
	15		0.51 ± 0.08 ^a^	48		15		0.50 ± 0.10 ^a^		48
	18		0.64 ± 0.11 ^a^	48		18		0.61 ± 0.12 ^a^		48

^1^ WAP: week after priming; ^2^ BTH: benzothiadiazole. Notes: the different letters indicate significant differences (ANOVA and EMMs post hoc; α = 0.05) between groups in the same week after priming for the corresponding parameter. n corresponds to the sample size of the previous parameters.

**Table A6:** Fruit compounds, displayed as the mean and standard deviation in early, intermediate, and late trusses per tomato plant, differentiated for the six treatments (part 1 of 2).

Treatment	Stage of Harvest	TCarC ^1^	TAC ^2^	TPC ^3^	FC_Catechin_ ^4^	FC_Quercetin_ ^4^	n
		[µg mg^−1^ DM^−1^]	[µg CyEs mg^−1^ DM^−1^]	[µg GAEs mg^−1^ DM^−1^]	[µg CEs mg^−1^ DM^−1^]	[µg QEs mg^−1^ DM^−1^]	
Non-treated + non-stressed (control)	early ^6^	0.2 ± 0.2 ^a^	1.1 ± 0.3 ^a^	4.8 ± 0.3 ^a^	3.5 ± 1.0 ^a^	2.7 ± 0.5 ^a^	16
	intermediate ^7^	0.3 ± 0.1 ^a^	0.8 ± 0.3 ^a^	5.2 ± 0.3 ^a^	3.8 ± 1.5 ^a^	2.5 ± 0.6 ^a^	16
	late ^8^	0.5 ± 0.2 ^a^	0.9 ± 0.3 ^a^	6.0 ± 0.5 ^a^	5.7 ± 1.8 ^a^	3.0 ± 0.7 ^a^	15
Non-treated + salt-stressed	early	0.2 ± 0.2 ^a^	1.1 ± 0.3 ^a^	4.9 ± 0.4 ^a^	3.3 ± 0.9 ^a^	2.6 ± 0.8 ^a^	16
	intermediate	0.3 ± 0.2 ^a^	1.0 ± 0.4 ^a^	5.3 ± 0.4 ^a^	4.0 ± 1.1 ^a^	2.6 ± 0.6 ^a^	16
	late	0.5 ± 0.1 ^a^	0.9 ± 0.4 ^a^	5.9 ± 0.5 ^a^	5.4 ± 1.9 ^a^	2.8 ± 0.8 ^a^	16
Primed + non-stressed	early	0.1 ± 0.4 ^a^	1.2 ± 0.2 ^a^	5.1 ± 0.3 ^a^	4.1 ± 0.8 ^a^	2.7 ± 0.4 ^a^	14
	intermediate	0.3 ± 0.2 ^a^	0.9 ± 0.3 ^a^	5.4 ± 0.3 ^a^	3.8 ± 1.4 ^a^	2.6 ± 0.5 ^a^	16
	late	0.5 ± 0.1 ^a^	1.0 ± 0.4 ^a^	5.9 ± 0.4 ^a^	6.3 ± 2.2 ^a^	2.9 ± 0.7 ^a^	16
Primed + salt-stressed	early	0.2 ± 0.2 ^a^	1.2 ± 0.3 ^a^	4.9 ± 0.3 ^a^	3.6 ± 0.8 ^a^	2.7 ± 0.3 ^a^	16
	intermediate	0.4 ± 0.1 ^a^	1.0 ± 0.3 ^a^	5.3 ± 0.5 ^a^	4.0 ± 0.8 ^a^	2.6 ± 0.4 ^a^	14
	late	0.5 ± 0.2 ^a^	0.9 ± 0.3 ^a^	6.0 ± 0.5 ^a^	4.9 ± 1.3 ^a^	2.7 ± 0.7 ^a^	16
BTH ^5^-treated + non-stressed	early	0.2 ± 0.2 ^a^	1.2 ± 0.2 ^a^	4.9 ± 0.4 ^a^	3.6 ± 0.6 ^a^	2.8 ± 0.5 ^a^	16
	intermediate	0.4 ± 0.2 ^a^	1.0 ± 0.4 ^a^	5.2 ± 0.4 ^a^	4.1 ± 1.1 ^a^	2.7 ± 0.6 ^a^	16
	late	0.4 ± 0.2 ^a^	0.8 ± 0.2 ^a^	5.8 ± 0.4 ^a^	4.9 ± 1.7 ^a^	2.6 ± 0.5 ^a^	15
BTH-treated + salt-stressed	early	0.2 ± 0.1 ^a^	1.1 ± 0.2 ^a^	4.7 ± 0.6 ^a^	3.3 ± 0.6 ^a^	2.5 ± 0.3 ^a^	16
	intermediate	0.3 ± 0.1 ^a^	1.0 ± 0.4 ^a^	5.2 ± 0.3 ^a^	3.8 ± 1.2 ^a^	2.6 ± 0.6 ^a^	15
	late	0.5 ± 0.1 ^a^	1.0 ± 0.3 ^a^	6.0 ± 0.7 ^a^	5.2 ± 1.8 ^a^	2.7 ± 0.5 ^a^	16

^1^ TCarC: total carotenoid content; ^2^ TAC: total anthocyanin content (expressed as CyEs, cyanidin-3,5-O-diglucosid equivalents); ^3^ TPC: total phenolic content (expressed as GAEs, gallic acid equivalents); ^4^ FC: flavonoid content (expressed as CEs, catechin equivalents, or QEs, quercetin equivalents); ^5^ BTH: benzothiadiazole; ^6^ early: 3rd truss per plant; ^7^ intermediate: 5th truss per plant; ^8^ late: 7th truss per plant. Notes: the different letters indicate significant differences (ANOVA and EMMs post hoc; α = 0.05) between groups with the same stage of harvest for the corresponding parameter. n corresponds to the sample size of the previous parameters.

**Table A7:** Fruit compounds, displayed as the mean and standard deviation in early, intermediate, and late trusses per tomato plant, differentiated for the six treatments (part 2 of 2).

Treatment	Stage of Harvest	Brix	TA ^1^	n
		[°Bx]	[µg CAEs mg^−1^ DM^−1^]	
Non-treated + non-stressed (control)	early ^3^	4.2 ± 0.3 ^a^	5.0 ± 4.2 ^a^	16
	intermediate ^4^	4.2 ± 0.3 ^a^	1.9 ± 0.2 ^a^	16
	late ^5^	4.3 ± 0.3 ^a^	2.0 ± 0.2 ^a^	15
Non-treated + salt-stressed	early	4.1 ± 0.2 ^a^	5.6 ± 4.3 ^a^	16
	intermediate	4.1 ± 0.3 ^a^	2.3 ± 1.6 ^a^	15
	late	4.2 ± 0.2 ^a^	1.9 ± 0.1 ^a^	16
Primed + non-stressed	early	4.1 ± 0.2 ^a^	7.0 ± 4.1 ^a^	16
	intermediate	4.2 ± 0.3 ^a^	2.4 ± 1.8 ^a^	16
	late	4.0± 0.3 ^a^	1.9 ± 0.2 ^a^	14
Primed + salt-stressed	early	4.1 ± 0.5 ^a^	5.9 ± 4.5 ^a^	16
	intermediate	4.4 ± 0.3 ^a^	2.1 ± 0.2 ^a^	16
	late	4.1 ± 0.3 ^a^	1.9 ± 0.1 ^a^	16
BTH ^2^-treated + non-stressed	early	4.2 ± 0.3 ^a^	6.5 ± 4.2 ^a^	16
	intermediate	4.2 ± 0.4 ^a^	2.0 ± 0.2 ^a^	16
	late	4.1 ± 0.4 ^a^	2.0 ± 0.2 ^a^	15
BTH-treated + salt-stressed	early	4.1 ± 0.3 ^a^	6.3 ± 4.3 ^a^	15
	intermediate	4.4 ± 0.4 ^a^	2.0 ± 0.2 ^a^	16
	late	4.2 ± 0.4 ^a^	2.0 ± 0.3 ^a^	16

^1^ TA: titratable acidity (expressed as CAEs, citric acid equivalents); ^2^ BTH: benzothiadiazole; ^3^ early: 3rd truss per plant; ^4^ intermediate: 5th truss per plant; ^5^ late: 7th truss per plant. Notes: the different letters indicate significant differences (ANOVA and EMMs post hoc; α = 0.05) between groups with the same stage of harvest for the corresponding parameter. n corresponds to the sample size of the previous parameters.
